# The Determinants of Adoption and Intensity of Climate-Smart Agricultural Practices among Smallholder Maize Farmers

**DOI:** 10.3390/su142416926

**Published:** 2022-12-16

**Authors:** Khethiwe Naledi Mthethwa, Mjabuliseni Simon Cloapas Ngidi, Temitope Oluwaseun Ojo, Simphiwe Innocentia Hlatshwayo

**Affiliations:** 1 Department of Agricultural Extension and Rural Resource Management, School of Agricultural, Earth and Environmental Sciences, College of Agriculture, Engineering and Science, University of KwaZulu-Natal, Private Bag X01, Scottsville, Pietermaritzburg 3201, South Africa; 2 Centre for Transformative Agricultural and Food Systems, School of Agricultural, Earth and Environmental Sciences, College of Agriculture, Engineering and Science, University of KwaZulu-Natal, Private Bag X01, Scottsville, Pietermaritzburg 3201, South Africa; 3 Department of Agricultural Economics, Obafemi Awolowo University, Ile-Ife 220101, Nigeria; 4 Disaster Management Training and Education Centre for Africa, University of the Free State, Bloemfontein 9301, South Africa; 5 African Centre for Food Security, School of Agricultural, Earth and Environmental Sciences, College of Agriculture, Engineering and Science, University of KwaZulu-Natal, Scottsville, Pietermaritzburg 3201, South Africa

**Keywords:** climate change impact, climate-smart agriculture (CSA), CSA adoption, smallholder farmers

## Abstract

Smallholder farmers’ maize production is highly susceptible to climate change. Higher temperatures may result in reduced yields while encouraging weed, pest, and disease infestation. The impacts of climate change on agriculture are projected to be negative, threatening global food security; therefore, this is an important area of empirical research. This study sought to identify the factors affecting the adoption and intensity of climate-smart agriculture (CSA) adoption among smallholder maize farmers in the uMshwathi and uKhahlamba Municipal areas of KwaZulu Natal. The study used the primary data collected from 99 respondents who were selected through simple random sampling. The descriptive results indicated that farmers had experienced severe climatic conditions such as drought, pests, diseases, hailstorms, heavy rains (floods), soil infertility, and frost in their farming system. The first hurdle of the probit model results showed that drought, on-farm income, and household size significantly influenced the adoption of CSA practices. In contrast, the main source of income and educational level had a significant negative influence. The results from the second hurdle showed that drought had a significant positive impact on the intensity of CSA adoption, while marital status had a significant negative effect. Several factors influence the adoption of an intensification of CSA practices. The study recommends that policymakers and climate change champions consider smallholder farmers’ socioeconomic factors when developing climate change adaptation programs. Local climate change organizations must scale up climate change awareness and adaptation programs collaboratively. Investments in public climate and adaptation education or training are needed, as well as localized meteorological observations and early warning systems. Mass media dissemination of climate change and adaptation information in locally understood languages is urgently required.

## 1 Introduction

Smallholder agriculture has been identified as a mechanism for achieving rural development goals in South Africa [1], and also for achieving favorable outcomes for food and nutrition security. Smallholder farmers are the drivers of many economies; therefore, climate change’s adverse effects can have undesirable results for the affected countries and farming households. They can play an essential role in livelihood creation among the rural poor in Africa, particularly in South Africa. Even though smallholder farmers have this potential, they are characterized by limited land access, capital, technologies, and market orientation [2,3]. Moreover, smallholder farmers are more susceptible to climate change as they have poor managerial production skills and limited access to quality irrigation systems. Therefore, these characteristics of smallholder farmers lead them to rely more on stable crops, such as maize, beans, and sweet potatoes, because these crops can be grown under unfavorable conditions [4]. Several smallholder farmers depend on maize (*Zea mays* L.) as one of the essential sources of food and nutrition [5,6]. An estimated one million smallholder farmers are growing maize for subsistence and local consumption [7,8]. However, extreme climate changes cause a negative impact on the production of maize because it is sensitive to rising temperatures and erratic rainfall patterns. High temperatures and drought conditions negatively impact the production, flowering, and yields of maize [7]. Building resilient agriculture for smallholder farmers requires adopting climate-smart agriculture (CSA), which can help them cope with the impact of climate change.

The Food and Agriculture Organization (FAO) [9] indicated that agriculture must be “climate smart” to feed the world in a way that can ensure sustainable rural development. CSA is an approach by which to develop long-term agricultural development conditions for food security in the context of climate change [9]. It contributes to national food security and development goals by focusing on three objectives including increasing agricultural productivity and incomes, increasing adaptability and resilience to climate change, and reducing or eliminating greenhouse gas emissions [9]. Several studies [10,11] reported that adopting several CSA practices helps improve food security. These studies further explained that adopting numerous CSA practices helps mitigate the impact of climate change and enhance nutrient availability. Antwi–Agyei et al. [11] revealed that most farmers use CSA practices to reduce pests and diseases, obtain higher yields and increase income. However, a primary concern of CSA technologies is adoption. Most CSA technologies are costly and out of reach for many smallholder farmers in the country [12–14]. Some difficulties include insufficient institutional support, insurance plans, and funding mechanisms, and unreliable legal frameworks, such as tenure rights and land management frameworks, which might reduce farmers’ willingness to embrace CSA [8,15,16]. CSA has been documented as a well-recognized method for adjusting agricultural output to the new conditions brought on by climate change in the literature. Although CSA practices are recommended to solve these problems, improve sustainable agriculture, and reduce poverty, it has been emphasized that smallholder farmers find it challenging to adopt and apply CSA-related technology.

Previous studies reported that socioeconomic factors such as age, education, farm size, and farm ownership affect whether smallholder farmers can effectively implement CSA [3,16,17]. These socioeconomic characteristics are also expected to impact the probability of CSA adoption and the number of CSA used by the farmers in this study. Generally, studies on adopting CSA practices in South Africa concentrate on factors affecting a specific CSA practice [4,18]. For instance, Abegunde et al. [19] investigated the factors that affect the adoption of CSA practices in smallholder farming households. However, the study did not differentiate between long-term and short-term adaptation strategies and did not focus on a specific crop. Farmers are often presented with various technologies that can be used as complements or substitutes to mitigate and adapt to climate change when planting certain crops. Therefore, there is a need for a deeper understanding of whether these factors influence CSA implementation in KwaZulu Natal maize farming because maize is a common and stable crop for smallholder farmers. This study set out to investigate the factors affecting the adoption and intensity of the adoption of CSA practices by maize farmers in the study areas. Furthermore, the study also seeks to understand the different types of short-term and long-term CSA practices adopted by smallholder farmers to mitigate climate change impacts. The study structure consists of five sections, including this introductory section. The second section discusses the methodology used for this study, which includes a description of the study area, data collection methods, and conceptual framework. The third section summarizes the research findings, focusing on two key themes: the determinants of CSA adoption and the intensity of CSA adoption among smallholder farmers. The fourth section discusses the findings of interviews, focus groups, and empirical frameworks. Finally, the conclusion provides a brief summary and critique of the findings and areas for future research.

## 2 Methodology

### 2.1 Description of the Study Area

The study was carried out at Swayimane and Bergville, located in KwaZulu Natal Province. Farmers rely on maize annually for their household consumption, animal feed, and source of income in these villages. These areas were also chosen because of farmers’ long relationships with active agricultural organizations from the public, private, and NGO sectors. Swayimane is in ward 8, and UMshwathi, uMgungundlovu is in KwaZulu Natal. The site covers an area of 32 km^2^. The area’s population is 6856 (53% female), consisting of 100% black Africans speaking IsiZulu as a home language. Most families are female-headed, and the livelihoods are primarily derived from subsistence farming [20]. Farmers commonly grow maize, beans, taro (Amadumbe), sweet potatoes, and sugarcane. The area comprises good rainfall (500 to 800 mm yr^−1^), predominant fog, and deep soils. Because the community is located in the uMgungundlovu District Municipality, it was projected to be prone to a warmer future and unpredictable changes in annual rainfall, floods, and storms. The impact of climate change projected is already experienced in the community [21]. Farmers in this community form cooperatives, farmers’ groups, and hold monthly meetings to exchange important information relevant to production and empower each other. However, they mostly prefer to work in their fields and generate income individually.

**Figure 1:**
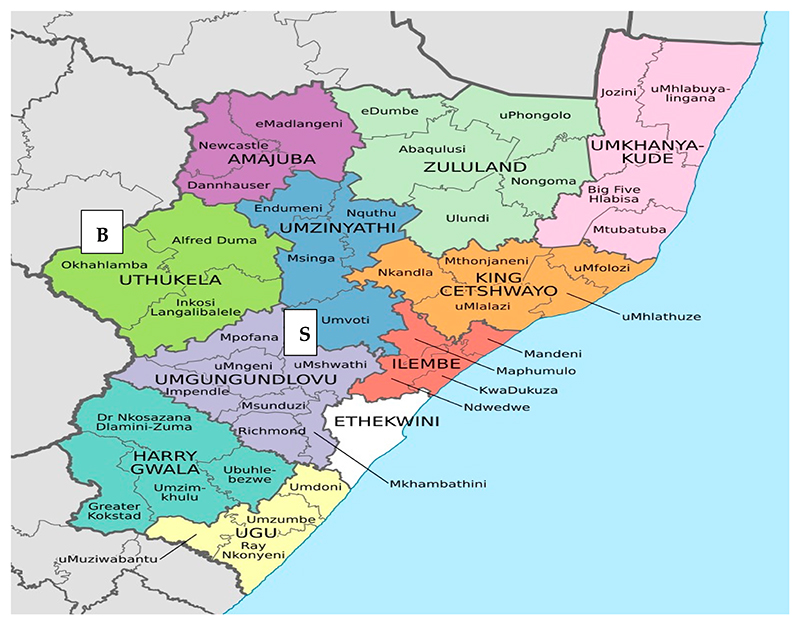
The map showing KZN Map with uMgungundlovu ad UThukela District Municipality Source: https://List_of_municipalities_in_KwaZulu_Natal (accessed on 20 August 2022). Note: B represent Bergville, S represent Swayimane.

Bergville is a small town in the UThukela District Municipality in KwaZulu Natal (Figure 1). It is situated in the foothills of the Drakensberg Mountains under the uKhahlamba local municipality. The number of people in Bergville is approximately 4500. Though Bergville is such a small town, the birth rate or population growth increases daily, increasing poverty. The job opportunities are very few, most people of Bergville are illiterate as a result, and most people make a living by engaging in farming. Farming ends up being their only hope for survival. The area has an average annual precipitation of 895 mm, where the rainy season falls in January, February, March, November, and December. Dry periods are in May, June, July, and August. January is the wettest month, with 154 mm of precipitation. On average, June is the driest month, with 11 mm of rainfall. It is almost impossible to produce crops in dry, cold winters with frost. Therefore, production is limited to summer [22].

### 2.2 Data Collection Method

The study adopted both qualitative and quantitative approaches. This study used the primary data collected by using semi-structured interviews and questionnaires. A total of 99 respondents were interviewed and randomly selected (49 from Swayimane and 50 from Bergville). A semi-structured interview with open-ended questions was administered through focus group discussions (FGDs) to find out about the farmer’s short-term adaptive measures for the 2019/2020 maize production season. These semi-structured interviews focused on analyzing which natural hazards threaten farmers’ maize yields, how they cope with them, and which potential practices are required in their farming system to increase their production. In addition, a questionnaire was given to find the long-term adaptation strategies. A total of 29 types of practices were identified with the assumption that each can deliver one or more CSA goals. These practices were grouped into five categories. These categories are knowledge smart, carbon smart, nitrogen smart, water smart, weather smart, and energy smart. Adoption of practices was self-reported in response to yes/no questions. Table 1 shows the 29 CSA practices and their categories identified in the study area.

### 2.3 Conceptual Framework

#### 2.3.1 The Determinants of CSA Adoption by Smallholder Maize Farmers

The adoption of CSA practices can be outlined as the stage at which a farmer decides to adopt one or more adaptive options to mitigate the effect of climate change. The double-hurdle model framework includes the first-stage adoption of climate change adaptation strategies based on the same set of covariates, determining the intensity of CSA adoption. The standard errors from separate estimations are valid for statistical inference if the error terms in the equations are assumed to be uncorrelated-conditional on all covariates. If there is no assumption of the error terms in the equation, the coefficient estimates from separate regressions will be biased [5,23]. The same method used by Heckerman to test bias is used for testing conditionally uncorrelated errors. However, it is standard to enforce one justifiable exclusion restriction estimating the second stage but not technically necessary for identification. The coefficient estimates on inverse mill ratio (IMR) test the null hypothesis that the first- and second-stage errors are conditionally uncorrelated. The model must be estimated and estimated again to conduct valid inference unless the coefficient estimates are statistically significantly different from zero [24]. The second-stage parameters are estimated without the IMR if there is a failure in rejecting the null [25].

A probit model of CSA for selection equations is estimated by using a function of explanatory variable that is also likely to determine the CSA intensity. The IMR predicted by the first-stage probit regression is used as a regressor in the second hurdle to account for selection bias. The probit regression model has been used by many researchers in studies relating to adoption. Serote et al. [26] used the probit regression model to evaluate the factors influencing the adoption of climate smart irrigation technologies for sustainable crop productivity by smallholder farmers. Mutenje et al. [27] used the probit regression model to evaluate the cost-benefit analysis of CSA options in southern Africa. They were balancing gender and technology. Marenya et al. [28] used the probit regression model to predict minimum tillage adoption among smallholder farmers. In contrast, Ojo et al. [29] used this model to evaluate the determinants of climate change adaptation strategies and their impact on the net farm income of rice farmers in southwest Nigeria.

According to Feder et al. [30], to determine the probability of CSA adoption by small-holder farmers, the underlying latent variable that captures the true farmer’s socioeconomic characteristics is hypothesized. The regression Equation (1) indicates the latent variable CSA^*^_i_:

(1) $$\begin{array}{c} CSA^{*}i=X_{i}\beta +ei\;ei\approx Y(1)\\ \;(\text{first hurdle})\; \end{array}$$

(2) $$\begin{array}{l} CSA^{*}i=1\;if\;CSA^{*}i>0\\ CSA^{*}i=0\;if\;CSA^{*}i\leq 0. \end{array}$$

CSA^*^_i_ has a value of 1 if a smallholder farmer adopts climate change and 0 otherwise. A vector to be estimated is *β*. A probit model of CSA^*^_i_, which follows random utility, is expressed as in Equation (2) [24]; a probit model of CSA which follows random utility is defined in Equation (3):

(3) $$\text{Hr}\;\left(CSA_{i}=1|B_{i},\alpha \right)=\oint \left(B_{i},\alpha \right)+ei.$$

CSA^*^_i_ equals 1 for households that adopt climate-smart agriculture techniques and 0 otherwise. Xi represents the vector of independent variables, *α* is the vector of parameters to be estimated. The *φ* is the standard normal cumulative distribution function, and *ei* is a random error term hypothesized to be distributed generally with unit variance and zero means.

#### 2.3.2 The Intensity of CSA Use among Smallholder Maize Farmers

The Poisson regression model (PRM), the negative binomial regression model (NBRM), the zero-inflated Poisson (ZIP), and the zero-inflated negative binomial (ZINB) are the most commonly used regression models to analyze count data. The PRM and NBRM regression models are standard for analyzing the response variables with nonnegative integers [31,32]. The last two (ZIP and ZINB) are commonly used for cases with frequent zero counts. This study does not have more zeros than would be expected. Therefore, only the PRM is discussed because the response variable contained nonnegative integers with only a few zero counts. A Poisson model was used to estimate the determinants of CSA intensity. Poisson is a process of satisfying the assumptions of the probability distribution of the number of occurrences of the event in a fixed time interval [33]. Studies by Ojo et al. [29] and Bukchin et al. [34] have stated that farmers usually weigh the technology’s benefits before adopting it. The study used the PRM because diagnostic tests revealed the absence of overdispersion and under dispersion. The density function of the PRM, as depicted in Equation (4), is given by

(4) $$\text{Hr}(P=p)=\frac{e^{-\delta (P)}\delta i{\left(p\right)}^{P}}{\text{Φ}(1+P)},$$

where *δ*_*i*_ = Exp (Ω +*B*^*i*^*ψ*) and *P*_*i*_ = 0, 1, …, *i* is the number of CSA practices used by the farmers, and B is a vector of predictor variable Ω and is Ψ the parameters to be estimated. The expected outcome is the number of CSA practices adopted by a farmer, expressed in Equation (5):

(5) $$\text{A}\left(P_{i}=p_{i}\right)=\text{Kar}\left\{\frac{P_{i}}{p_{i}}\right\}=\delta_{i}=\text{Exp}\left(\text{Ω}+B^{i}\text{Ψ}\right)\;\text{for}\;i=1,2,\ldots ,n.$$

## 3 Description of Variables and Statistics

The quantitative data analysis of the study was conducted by using the IBM Statistical Package for Social Science (SPSS) version 28. The descriptive statistics provided the key socioeconomic characteristics of sampled smallholder farmers. According to the hypothesis of this study, the variables are drought, gender, marital status, once-off-farm income, smartphone, access to market information, membership in farmers’ association, the primary source of income, household size, age, and level of education, and these variables affect the adoption and intensity of adoption of CSA practices. Table 2 summarizes the descriptive variables’ names, variable types, measurements, and expected outcomes.

## 4 Results

### 4.1 Descriptive Analysis of the Results

#### 4.1.1 Demographic Characteristics of Smallholder Farmers in the Study Area

Table 3 shows that 80% of the respondents were female, and 20% were male. The results show that most (42%) respondents had received primary education, followed by 33% of the respondents who had no formal education, 24% who had received secondary education, and only 1% who had acquired tertiary education. The results also show that most (72%) farmers are married, 19% are single, 8% are widowed, and only 1% are divorced.

The results showed that approximately 39% of the respondents relied on pension grants, 38% relied on social government grants, and 8% relied on agricultural produce sales as their primary income source. Approximately 1% of the respondents had no source of income. When considering the second source of income, results show that the majority of the farmers (73%) did not have a second source of income. Approximately 21% indicated that their alternative income source was selling agricultural produce, and 3% owned spaza shops as an additional source of income. The results also revealed that household size was an average of 7, ranging between 1 and 30 members per household. Furthermore, the average age of participants was 56 years. This is in line with the literature, which reported that most farmers are older.

#### 4.1.2 Climate Change Impact (Natural Hazards) Experienced by Farmers in Their Maize Production in 2019–2020

**Figure 2:**
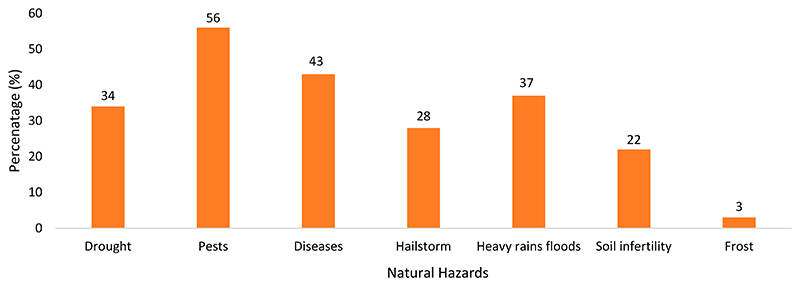
Natural hazards affecting maize production (Source: Own source).

Figure 2 shows the different natural hazards that affected farmers during the 2019–2020 production season of maize. These natural hazards affected mostly the quality and quantity of maize yields. The most common natural hazards that farmers experienced were pests, with 56% of the farmers experiencing this hazard, followed by diseases, with 43% of the farmers having experienced diseases in their maize crops. Approximately 34% of the farmers were affected by drought. Only 22% were affected by soil infertility. Farmers also reported that their yields decreased through predators such as livestock, monkeys, rats, and birds.

#### 4.1.3 Short-Term CSA Practices Adopted by Smallholder Maize Farmers to Cope with Natural Hazards in the 2019–2020 Season

**Figure 3:**
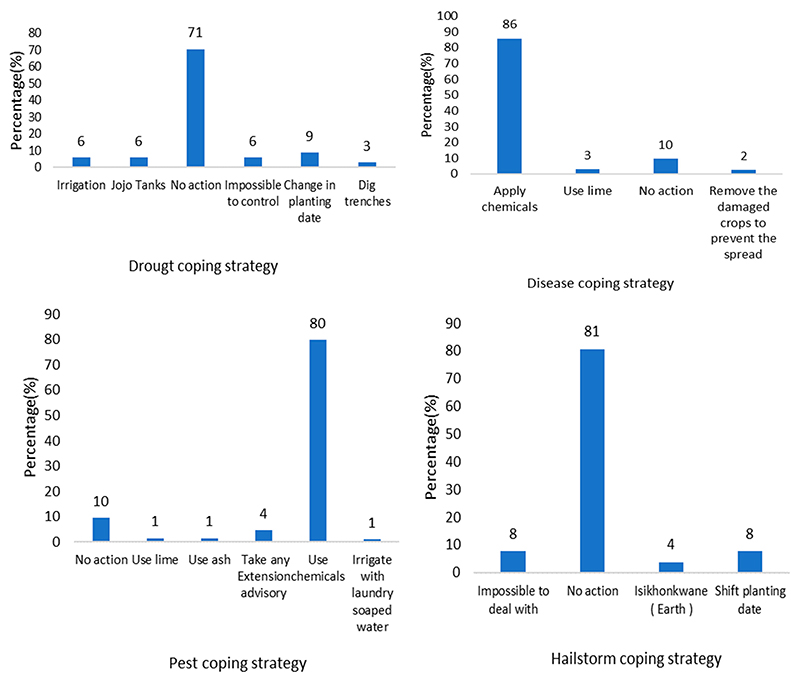
Short-term CSA practices adopted by smallholder maize farmers to cope with natural hazards in the 2019–2022 season.

Figure 3 shows the different short-term CSA practices adopted by smallholder maize farmers to cope with natural hazards in the 2019–2020 production season. The results showed that approximately 71% of the farmers did not take any specific action to cope with the drought. In comparison, 6% thought it was impossible to take measures to deal with the water shortage, and only 1% used in-field rainwater-harvesting methods by which farmers dig rows (trenches) in the soil to absorb rainwater. Regarding disease coping strategy, approximately 86% of the farmers applied chemicals to deal with diseases, 10% took no action, and only 2% removed the affected leaves or crops to prevent the spread of the disease. The results also showed that 80% of the farmers used chemicals to deal with pests, 10% took no action to adapt to pests, and 1% used greywater harvesting to cope with the problem—specifically, water with soap previously used for laundry.

Regarding the hailstorm adaptation strategy, approximately 81% of farmers took no action to adapt to hailstorms, 8% thought it was impossible to deal with hailstorms, and 8% shifted planting dates. Few farmers (4%) mentioned using “Isikhonkwane” meaning earth, to adapt to a hailstorm. Regarding the flood adaptation strategy, about 15% of the affected farmers did not take any actions to deal with floods, 2% dig trenches to channel water away from the plots, and only 1% thought it was impossible to adapt to floods. The results also showed that 3% of farmers were affected by frost, and all of them preferred planting earlier than usual so the crops could mature enough to withstand frost. As mentioned earlier, farmers reported that their yields are decreasing because of predators such as livestock, monkeys, rats, and birds. They are not putting any adaptation strategies to deal with predators except applying poison to rats.

#### 4.1.4 Potential Practices Which Farmers Can Put in Place to Adapt to Climate Change

**Figure 4:**
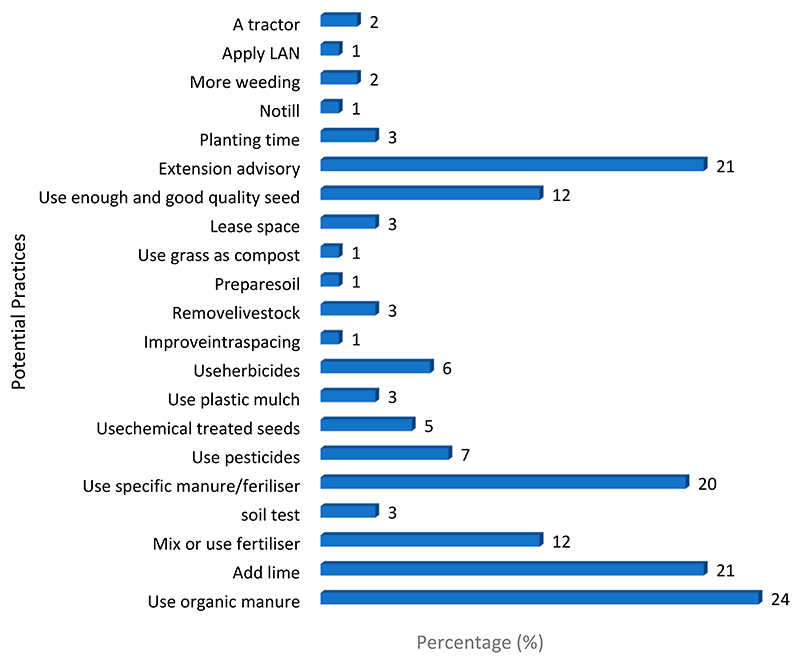
Potential practices to cope with hazards (Source: Own source).

Figure 4 shows the potential practices which farmers believe could be an aid to withstand natural hazards and increase production. The most preferred potential practice was the use of organic manure 24%. Approximately 21% of farmers prefer to add lime to neutralize soil PH, mix different fertilizers, and rely on extension advisory services, whereas 20% prefer using specifically recommended fertilizers.

#### 4.1.5 The Adaptation Strategies Employed by Smallholder Farmers over Ten Years of Maize Production

**Figure 5:**
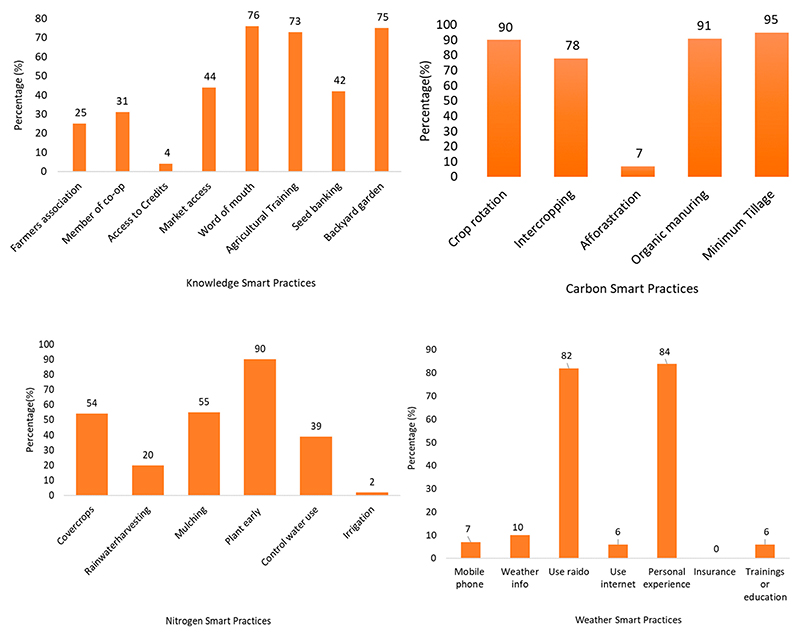
Long-term climate-smart practices adopted by sampled smallholder maize farmers.

Figure 5 shows the long-term CSA practices employed by smallholder farmers as adaptation strategies over a long-term period of more than ten years of maize production. The results show that as far as knowledge of Smart practices is concerned, the most adopted practice is sharing one-on-one information with colleagues, also known as word of mouth or farmer-to-farmer knowledge sharing, at 76%, and only 4% had access to agricultural credits. Regarding carbon-smart practices, 95% have adopted minimum tillage, followed by 91% of farmers who used plant and animal manure, also known as organic manure, and 90% used crop rotation. Approximately 78% of farmers produced different kinds of crops together on the same ground, also known as intercropping. The practice that farmers least adopted was afforestation; only 7% of farmers planted trees around their fields. Regarding nitrogen-smart practices, the results showed that most farmers (77%) preferred to plant legumes to improve soil fertility, 59% estimated the amount of fertilizer required, and only a few farmers (25%) used specific fertilizer.

The common practice farmers have adopted regarding water-smart practices is planting early in a season (90%) to use rainwater efficiently, followed by 54% of farmers who planted cover crops to maintain soil moisture, and 55% who used mulch to reduce excessive use of water. Only 2% of the farmers use irrigation. Among weather-smart practices, about 43% of farmers used personal experience to predict weather events, and 42% used radio or TV for weather information. The results show that no farmers take index-based insurance to protect their crops from weather events. Regarding energy-smart practices, most farmers (74%) used compost.

#### 4.1.6 Determinants of Adoption of Climate Change Adaptation Strategies among Maize Farmers Probit Model

Table 4 highlights the determinants of adopting climate change adaptation strategies among smallholder farmers in the sampled areas. The first-hurdle equation of a probit regression model revealed that drought, on-farm income, the main source of income, household size, and level of education were the main factors that significantly affected the adoption of CSA practices by smallholder farmers.

The results revealed that drought had a positive and significant impact on the adoption of CSA. On-farm income showed a positive and statistically significant (*p* < 0.05) effect on the adoption of CSA among smallholder farmers. Unexpectedly, the results showed that the main source of income had a negative and significant impact on CSA adoption. Household size positively impacted CSA adoption and was statistically significant at the 5% level. Surprisingly, the results showed that education level negatively impacted the adoption of CSA practices methods and was statistically significant.

#### 4.1.7 Determinants of the Intensity of CSA Adoption

The results of the intensity of CSA adoption are presented in Table 5. An inverse Mills ratio (IMR) predicted from the first-hurdle equation was used as a covariate in the count data model (second hurdle of the Poisson model) to correct for selectivity bias. The results show that the IMR was statistically significant, indicating that selection bias was a problem. Because the coefficient was significant, the null hypothesis (no selection bias) is rejected. Hence, using a double-hurdle model for estimating determinants and level of adoption of CSA while correcting for a selection bias problem is justified. As shown in Table 5, the estimation of Akaike information criterion (AIC) and Bayesian information criterion (BIC) are essential to indicate a better model in analyzing count data of the level of adoption of CSA of smallholder farmers. In this study, the Poisson regression model was used, the AIC value was 362.705, and the BIC value was 396.442. Surprisingly, a Poisson model revealed that only two variables significantly impacted the intensity of CSA adoption. Drought and marital status were the main factors that significantly affected the intensity of CSA adoption by smallholder farmers in KwaZulu Natal.

Similar to the first double hurdle of the probit regression model, the second double hurdle of the Poisson regression model showed that drought positively influenced the intensity of CSA adoption by smallholder farmers and was statistically significant. The results also showed that a farmer’s marital status had a negative and significant relationship with the intensity of CSA adoption.

## 5 Discussion

This study aimed to assess the determinants of CSA adoption and the intensity of CSA adoption among smallholder maize farmers. The descriptive results indicated that farmers had experienced severe climatic conditions such as drought, pests, diseases, hailstorms, heavy rains (floods), soil infertility, and frost. This proves that climate change is still a major agricultural production issue, especially within smallholder farming. These results align with most previous studies’ findings that agriculture is prone to harsh climatic conditions [3,35,36].

The empirical results revealed a positive relationship between drought conditions and CSA adoption and the intensity of CSA adoption. Rainfall, dams/rivers, communal taps, wells, and boreholes are the primary water sources for most smallholder farmers. However, due to varying rainfall amounts, some water sources are seasonal, making it difficult for rural farmers to produce annually [26]. These results imply that farmers go the extra mile searching for different practices to put in place to save and harvest water to cope during water-shortage seasons. The results seem plausible to findings by Kanjere et al. [37], who emphasized that farmers need to adopt CSA practices to help access and maintain water to adapt to water shortages. On the contrary, Negera et al. [38] found that farmers who have previously been affected by drought are less likely to adopt and intensify CSA practices because the cost of adopting CSA practices is higher when farmers experience crop failure due to severe climatic conditions.

On-farm income in this study refers to the revenue farmers generate through making sales of their agricultural produce. The positive relationship between on-farm income and CSA adoption revealed in this study implies that farmers who sell their products to make income adopt CSA practices more than farmers who grow primarily for household consumption and those who generate income outside farming. Similarly, the study by Adeagbo et al. [5], Onyeneke et al. [39] found that farmers who sell their produce invest a lot in adaptation practices with the mandate to increase production. Furthermore, Asrat et al. [23] stated that farmers who obtain off-farm income are less likely to invest in adaptation methods because they are not solely dependent on farming.

This study’s findings indicate an adverse relationship between the main source of income and CSA adoption. This result is convincing, as most smallholder farmers’ main sources of income in the study area were old age and child support, indicating that the farmers’ ability to afford to invest in new technologies is low. Another possible explanation of the negative relationship could be based on the findings that only 8.1% rely on agricultural sales as their main source of income. The results indicate that farmers in this study are less likely to be adventurous in investing in CSA practices, as most do not expect any returns from production but only grow crops for household consumption. These results correspond to the studies of Onyeneke et al. [39], who found that only a few smallholder farmers are commercially orientated and take their produce to the market; hence, they are less likely to adopt CSA practices. Furthermore, Hlatshwayo et al. [40] stated that most households see social grants as their main source of income and neglect farming.

The study results show a positive relationship between adopting CSA practices and household size. These results imply that more people in the household means more labour and exposure to information sources and hence more ideas about climate change adaptation strategies. This is in line with Adeagbo et al. [5] and Agbenyo et al. [41], who found that household size positively influences the likelihood of CSA practices adoption. On the contrary, Musafiri et al. [42] found that large families were less likely to adopt labor-intensive CSA practices, and small family sizes adopted and hired labor to implement the innovations.

The educational level of the farmer decreased the adoption of CSA practices. The implication of results could be attributed to the fact that farmers with a low educational level have fewer information-comprehension skills, and therefore are less aware of climate change and, hence, less likely to respond to the effects of climate change. These findings are consistent with the findings of the Department of Agriculture [43], which stated that recent technological improvement and information necessitate a certain level of formal education and training because most technologies are frequently presented in complex academic language, making it difficult for illiterate farmers to use them. The findings by Kolawole et al. [16] and Salad et al. [44] stated that the more educated the farmer becomes, the more likely it is that they will use scientific weather knowledge in farming decisions. Again, farmers with low levels of formal education are less likely to adopt CSA practices because they cannot search, process, interpret, and respond to new information on CSA practices. Higher levels of education tend to build innovativeness and improve the farmers’ information processing, which is essential in the adoption of technological decision-making choices [45,46].

Surprisingly, the results of this study show that a farmer’s marital status has a negative relationship with the intensity of CSA adoption. The possible explanations could be that most married farmers with kids are more likely to invest in domestic work than in the garden, and the spouse opts for off-farm activities to sustain household livelihood. Therefore, there is less time and money invested in agricultural innovations. The findings of this study are consistent with previous studies that show that marital status increases access to alternative sources of income outside of farming, decreases the time and effort dedicated to farming activities, and decreases labor, endowment, and investment in adopting new technology [28,46]. Moreover, Negera et al. [38] found that a spouse’s education negatively influences the adoption of new technologies, as an educated individual prefers white-collar employment to sustain livelihood rather than sustaining livelihood via CSA adoption. The findings of this study are contrary to the results of [44]. They revealed that farmers’ marital status had a positive relationship with the intensity of climate-smart agriculture practices because marriage promotes family life by allowing both women and children to participate in crop production and technology use.

## 6 Conclusions and Policy Recommendations

The adoption of CSA practices not only helps smallholder farmers to increase their agricultural productivity but also helps to improve smallholder farmer resilience to the impact of climate change. This study assessed the factors affecting the adoption and intensity of CSA adoption among smallholder maize farmers. The findings showed that drought, on-farm income, and household size significantly influenced the adoption of CSA practices. In contrast, the main source of income and educational level had a significant negative influence. Drought also had a significant positive impact on the intensity of CSA adoption, whereas marital status had a significant negative effect. It is concluded that socioeconomic characteristics had a considerable influence on the likelihood of CSA adoption and the intensity of adoption by smallholder maize farmers. Improved education among smallholder farmers can improve CSA adoption. Word of mouth, by which farmers share information with their family and friends, significantly improved the knowledge about climate change and adaptation. The study recommends that numerous local organizations with a strong comparative advantage in agriculture, climate change, and adaptation collaborate to invest in increasing rural public awareness of climate change and adaptation. An investment in outcome-based accredited training that caters to all farmers’ primary education levels will ensure that training activities significantly impact the outcome of competent farmers with skills and knowledge about climate change and adaptation. The study suggests that climate change awareness and adaptation information be disseminated through the media and in locally understood languages. The study only looked at the factors that influence CSA adoption and intensity among smallholder maize farmers in one province of KwaZulu-Natal. Further research should be carried out in all nine provinces of South Africa.

## Figures and Tables

**Table 1:** The 29 CSA practices and their categories.

**Knowledge Smart**
Belong to farmer associations
Get access to information on market prices of produce & inputs
Share one-on-one information with colleagues (farmer-to-farmer knowledge sharing)
Store seeds for next season/emergency (seed banking)
Have a backyard garden in addition to my farm
**Carbon Smart**
Change the type of crop planted on this land in some seasons (crop rotation)
Plant different type of crops together (mix cropping)
Plant trees in and around my farm (afforestation)
Use plants and animal manure on my farm (organic manuring)
Use less heavy equipment on my farm (minimum tillage)
**Nitrogen Smart**
Use specific fertilizer/manure based on the type of soil (site-specific nutrient application)
Plant legumes among crops
Estimate the amount of fertilizer/manure needed at a time (precision fertilization)
**Water Smart**
Plant cover crops to maintain soil moisture
Harvest and store rainwater to be used on my farm
Engage in mulching to reduce excessive use of water
Plant in the early season to make use of rainwater
Regulate/control the water used in watering crops
**Weather Smart**
Use mobile phone to access weather information
Received weather information through the community information centre
Usage of radio/tv for weather information
Access to weather information on the internet
Use personal experience to predict weather events
Take index-based insurance (IBI) to protect my farm
Received education/training on how to access weather information by an organization
**Energy Smart**
Compost my residue after harvesting
Convert my residue into bioenergy
Use solar equipment in farming
Use of less fuel-consuming vehicles

**Table 2:** Description of variables (own source).

Variable	Description of Variable	Expected Outcome
Drought	Dummy (1 = Yes, 0 = otherwise)	+
Gender	Dummy (1 = Female, 0 = Male)	_
Marital status	Continuous (1 = Single, 2 = Married, 3 = Divorced, 4 = Widowed)	+
Once-off-farm income	Measured in Rands/Kg	_
Smartphone	Dummy ((1 = Owns, 0 = otherwise)	+
Access to market information	Dummy (1 = Yes, 0 = otherwise)	+
Member of farmers’ association	Dummy (1 = Yes, 0 = otherwise)	+
The primary source of income	Measured in Rands/Kg	+
Household size	Measured in numbers	+
Age	Measured in numbers	+
Level of education	Continuous (1 = No education, 2 = Primary, 3 = Secondary, 4 = Tertiary)	+

Source: Survey data.

**Table 3:** Demographic characteristics of smallholder maize farmers.

Variable	Description	Percent (%)
Gender	Female	80
	Male	20
Level of education	No Education	33
	Primary Education	42
	Secondary education	24
	Tertiary education	1
Marital status	Single	19
	Married	72
	Divorced	1
	Widowed	8
The main source of income	No income	9
	Agricultural produce	8
	Social government grant	38
	Remittances	1
	Pension	39
	Casual work	1
	Spaza	2
	Traditional healer	2
The second source of income	No income	73
	Full-time employment	1
	Part-time employment	1
	Sales of agricultural produce	21
	Casual work	1
	Spaza	3

**Table 4:** Probit regression of determinants of CSA adoption among the farmers’ first hurdle.

CSA Adoption	Coef.	St. Err.	*p*-Value
Drought	0.915	0.406	0.024 **
Gender	−0.509	0.438	0.245
Marital status	−0.414	0.259	0.110
On-farm income	0.000	0.000	0.050 *
Smartphone	0.043	0.487	0.929
Access to market info	0.170	0.376	0.652
Farmers’ association	−0.504	0.406	0.214
Main source of income	−0.269	0.116	0.021 **
Household size	0.109	0.053	0.040 **
Age	−0.003	0.014	0.823
Level of education	−0.521	0.244	0.033 **
Total anum	0.000	0.000	0.142
Constant	2.947	1.381	0.033 **
Pseudo r-squared	0.251		
Chi-square	33.712		
Akaike crit. (AIC)	126.597		
Bayesian crit. (BIC)	160.334		
Prob > chi2	0.001		

** *p* < 0.05, * *p* < 0.1.

**Table 5:** Poisson regression of determinants of intensity of CSA adoption among the farmers’ second hurdle.

CSA Number	Coef.	St. Err.	*p*-Value
Drought	0.731	0.190	0.000 ***
Gender	0.091	0.217	0.673
Marital status	−0.203	0.122	0.095 *
On-farm income	0.000	0.000	0.173
Smartphone	−0.097	0.263	0.713
Access to market info	−0.027	0.198	0.893
Farmers’ association	−0.084	0.229	0.714
Main source of income	−0.001	0.064	0.983
Household size	0.002	0.021	0.913
Age	0.002	0.007	0.736
Level of education	−0.044	0.135	0.741
IMR_01	2.280	0.559	0.000 ***
Constant	−0.752	0.881	0.394
Pseudo r-squared	0.181		
Chi-square	74.528		
Akaike crit. (AIC)	362.705		
Bayesian crit. (BIC)	396.442		
Prob > chi2	0.000		

*** *p* < 0.05, * *p* < 0.1.
